# On-Pump Beating Coronary Artery Bypass in High Risk Coronary Patients

**Published:** 2015-01

**Authors:** Abbas Afrasiabirad, Naser Safaie, Hosein Montazergaem

**Affiliations:** Department of Cardiac Surgery, Madani Heart Hospital, Tabriz University of Medical Sciences, Tabriz, Iran

**Keywords:** Cardio pulmonary bypass, Off pump coronary artery bypass, Efficacy

## Abstract

**Background:**

There are some conflicting results with Conventional Coronary Artery Bypass Grafts (CCABG) with arrested heart in coronary high-risk patients. Moreover, performing off-pump CABG in these cases may be associated with serious complications. The objective of this study is to evaluate the efficacy of the on-pump beating CABG (OPBCABG) in coronary high-risk patients in comparison with the conventional methods.

**Methods:**

In a prospective research study, 3000 off-pump CABG patients were considered during June 2003 to December 2011. Among these, 157 patients with one or more of the following risk factors were included for OPBCABG; severe left main stenosis, early post-acute myocardial infarction with ongoing chest pain, unstable angina, intractable ventricular arrhythmia, post complicated coronary intervention and severe left ventricular dysfunction. These patients were compared with 157 similar patients undergone CCABG with aortic cross clamp before 2003.

**Results:**

Preoperative patient characteristics revealed no significant differences between the two groups. The patients’ mean age and number of grafts were 57 years and 3 per patient respectively. Hospital mortality was 3.2% and 9% in OPBCABG and CCABG groups, respectively (P<0.001). Preoperative myocardial infarction, requirement of inotropic agents and intraaortic balloon pump, renal dysfunction and prolonged ventilation time were significantly higher in CCABG group.

**Conclusion:**

Our results suggest that OPBCABG is effective in coronary high-risk patients and significantly reduces mortality and the incidence of perioperative MI and other major complications.

## Introduction

Treatment strategies for ischemic heart diseases are complicated by the development of Off-pump coronary artery bypass (OPCABG) surgery and drug-eluting stents. Since percutaneous coronary intervention (PCI) is indicated in a wider range of cases and the remaining severe cases are subject to surgical revascularization, the best surgical approach should be selected carefully among OPCABG, OPBCABG, and CCABG. OPCABG, due to its advancement by improved surgical techniques and the development of new devices, is aggressively introduced even in high-risk patients (e.g. patients with severe calcification of the ascending aorta, elderly patients, and those complicated by cerebrovascular disorder, malignancy, multiple atherosclerosis, renal failure, and/or respiratory failure). Despite OPCABG advancement as a new procedure, on-pump beating and conventional CABG surgery remains as widely used revascularization approaches.^1^ Coronary high-risk cases usually require urgent surgical revascularization and some are hemodynamically unstable. Manipulation of the heart during OPCABG to perform distal anastomosis especially in the left circumflex territory, may lead to serious instability and complications. According to our experience and available literature,^2^^-^^5^CCABG on cardiopulmonary bypass with arrested heart in coronary high-risk patients is associated with high mortality and morbidity. To reduce the adverse effects of cardioplegia and global myocardial ischemia and ameliorate the hemodynamic effects of cardiac manipulation in OPCABG, we started on-pump beating heart coronary bypass in such a group of patients since 2003 as a newly offered technique to protect the heart. In this study, we assayed the efficacy of the OPBCABG in comparison with conventional techniques in coronary high-risk patients.

## Patients and Methods

We have started OPCABG since June 1999. Until August 2003, we performed CCABG with cold or tepid blood cardioplegia, by antegrade and retrograde route in high-risk coronary patients. Since then, we have switched to OPBCABG without aortic cross clamping in the following cases: severe left main stenosis, post-acute myocardial infarction, unstable angina, intractable ventricular arrhythmia patients, and severe left ventricular (LV) dysfunction. In a prospective research study between June 2003 and December 2011, 157 patients with one or more of the above-mentioned criteria were included. Cases at low or medium risk for CABG were excluded. These cases were matched with the same number of conventional method patients who had undergone CCABG before 2003 by constructing a propensity score from core patient characteristics. By introducing drug-eluting stents, PCI has become widespread in the treatment of coronary artery disease. Guidelines for coronary intervention were revised and emergency and high-risk CABG patients were significantly getting older with multiple co-morbidities. This was lacking in propensity score matching.

Operations were performed by two skilled coronary surgeons. Postoperative morbidity and mortality were evaluated during the 30 days after the operation. Postoperative MI and other complications were defined according to the guidelines. SPSS statistics 16.0 was used for statistical analysis and P>0.05 considered statistically significant.

*Surgical Techniques*

All procedures were performed through midsternotomy approach. After harvesting left internal mammary artery (LIMA) and other conduits, cardiopulmonary bypass (CPB) was established by cannulation of ascending aorta and right atrium. In CCABG, systemic temperature was maintained at 32^o^C and cardiac arrest was achieved with antegrade and retrograde cold or tepid blood cardioplegia in interval of 20 minutes to perform all distal anastomosis. After that, during the controlled retrograde blood perfusion, all proximal anastomoses were separately performed on the ascending aorta. In OPBCABG, distal anastomosis was performed by using suction stabilizer and intracoronary shunt and then proximal ones were connected to ascending aorta by utilizing aortic side clamp. LIMA to the left anterior descending (LAD) artery was the first anastomosis in many patients. In some patients with severe ischemia in the left circumflex or right coronary artery territory, after the completion of the distal anastomosis, conduits were connected to coronary octopus cannula (multiple perfusion adaptor, CalMed, California, USA) to maintain coronary flow during other anastomosis.

## Results

Demographic characteristics and operative data are shown in table 1. Preoperative patient characteristics revealed no significant difference between the two groups. CCABG patients required IABP more than OPBCABG and CPB time was also longer in this group.

**Table 1:** Demographic characteristics of the CABG patients and operative data

	**On-pump beating**	**Conventional**	**P value**
Age (years)	56.8±8	57.2±9	0.94
Gender (M:F)	2.9:1	3.1:1	0.59
LVEF (%)	38±9	34±8	0.24
CPB time (min)	86±23	115±28	<0.001
No. of grafts	3.1±0.4	2.9±0.5	0.82
LIMA use (%)	89	84	0.21
IABP (%)	6	13	<0.001

(M:F): Male to female; LVEF: Left ventricular ejection fraction; CPB: Cardio pulmonary bypass; LIMA: Left internal mammary artery; IABP: Intra-aortic balloon pump

As shown in table 2, postoperative renal failure, prolonged ventilation time, inotropes requirements, peak cardiac troponin I, and mortality were high in CCABG patients. Renal failure was defined to rising serum creatinine to higher than 1.5 mg/dl in the postoperative period. Prolonged ventilation time assigned to patients unable to be weaned from ventilator before 72 hours after operation. Patients who received full Dopamine and Dobutamine and required another inotropes, included in more inotropes need patient group. Severe left main coronary lesions were defined to patients with more than 70% stenosis. Prevalence of high-risk CABG patients is shown in table 3. Ongoing chest pain occurred in patients with unstable angina (U/A) and post myocardial infraction of those who were not suitable for PCI. Whenever the systolic left ventricular ejection fraction decreased to and below 35%, severe LV dysfunction was applied.

**Table 2:** Postoperative characteristics of the CABG patients

	**On-pump beating**	**Conventional**	**P value**
Renal failure	2.6%	6.4%	<0.02
Prolonged ventilation time	9.4%	16.8%	<0.001
Inotropes requirements	24%	36%	<0.001
Peak cardiac troponin I (ng/ml)	4±2	7±5	<0.05
Mortality	3.2%	9%	<0.001

**Table 3:** Prevalence of high-risk CABG patients

	**On-pump**	**Conventional**
U/A (unstable angina) (n)	(30) 19%	(52) 33%
Severe left main stenosis (more than 70%) (n)	(25) 16%	(28) 18%
Intractable ventricular arrhythmia (n)	(11) 7%	(13) 8%
Early post-acute MI (ongoing chest pain) (n)	(8) 5%	(11) 7%
Post PCI complication (n)	(19) 12%	(7) 4%
Severe LV dysfunction (less than 35%) (n)	(64) 41%	(47) 30%

MI: Myocardial infarction; PCI: Percutaneous coronary intervention; LV: Left ventricle

## Discussion

In the present study, it has been revealed that OPBCABG significantly reduced the duration of CPB and peak creatinekinase and troponin I (P<0.05). In OPBCABG, cross clamping of the aorta was eliminated, but in the conventional method, introducing global ischemia and protection of the heart by retrograde and antegrade cardioplegia is a routine manner. One of the challenging issues in the high-risk coronary patients is the protection of the heart during the surgery. For example, many of the diabetic patients suffer from multiple and diffuse coronary artery disease and fewer patients with acute post MI or severe left main lesions are in homeostatic derangement in cellular level.^6^^,^^7^ It is believed that, current cardioplegic techniques may not consistently avoid myocardial ischemic damage during global ischemia in these high risk patients undergoing CABG and may leads to difficult CPB wean off and prolonged time.^8^ We assume a low level of cardiac enzymes in OPBCABG derived from good cardiac protection by eliminating cross clamp of the aorta. In the recent years, the number of U/A patients decreased and post PCI complicated cases increased. Because PCI was expanded to multi-vessel coronary artery disease, thus post PCI early and late complications needing CABG were increased. In addition, severe left ventricular dysfunction cases during this time were raised and, in some ways, it may be related to stent complications.

The number of patients requiring additional intra-aortic balloon pump (IABP) support was significantly lower in the OPBCABG group. IABP can be used in many groups of patients, including unstable patients, early post MI, lower LVEF, intractable arrhythmia pre and post operation. Myocardial protection in these cases with low contractile reserve is still a surgical dilemma. Preoperative MI and postoperative low output syndrome are serious complications in such cases.^9^ In addition to the adequate protection of the heart in this situation, on-pump beating provides a condition to perform a complete revascularization and it may be justified to decline inotropes and IABP support.^10^^-^^12^

We showed that renal failure and prolonged ventilation were less in the OPBCABG. CPB per se might have damaging properties on organs including myocardium. CPB can trigger intense inflammatory response during and immediately after the operation.^13^ For this reason, some prefer OPCABG when technically feasible in some high-risk patients.^14^ Despite the damaging effect of CPB, preservation of the left ventricular contractility and maintaining perfect cardiac index may explain the drop-off in cardiac enzymes and improvement of organ function in on-pump CABG patients.^15^ Some patients in CCABG weaned off CPB with difficulty and experienced low cardiac output after operation, so it may justify a reverse finding. OPBCABG can be extended to hemodialysis patients not suitable to OPCABG.^16^ The use of CPB and the elimination of cardioplegic arrest may be beneficial for the short-term survival of chronic hemodialysis patients.

The main limitation of this study is related to the fact that it dealt with the historical match of the patients as well as its retrospective nature. 

## Conclusion

OPBCABG with a normothermic cardiopulmonary bypass was an effective method and is associated with improved hospital outcome. This could be the method of choice for some high-risk coronary patients. 

## References

[1] Varghese D, Yacoub MH, Trimlett R, Amrani M (2001). Outcome of non-elective coronary artery bypass grafting without cardio-pulmonary bypass. Eur J Cardiothorac Surg.

[2] Angelini GD, Taylor FC, Reeves BC, Ascione R (2002). Early and midterm outcome after off-pump and on-pump surgery in beating heart against cardioplegic arrest studies (BHACAS 1 and 2): a pooled analysis of two randomized controlled trials. Lancet.

[3] Bittner HB, Savitt MA (2002). Off-pump coronary artery bypasses grafting decreases morbidity and mortality in a selected group of high-risk patients. Ann Thorac Surg.

[4] D’Ancona G, Karamanoukian H, Kawaguchi AT, Ricci M, Salerno TA, Bergsland J (2001). Myocardial revascularization of the beating heart in high risk patients. J Card Surg.

[5] Goetze JP, Christoffersen C, Perko M, Arendrup H, Rehfeld JF, Kastrup J (2003). Increased cardiac BNP expression associated with myocardial ischemia. FASEB J.

[6] Srinivasan AK, Grayson AD, Fabri BM (2004). On-pump versus off-pump coronary artery bypass grafting in diabetic patients: a propensity score analysis. Ann Thorac Surg.

[7] Izumi Y, Magishi K, Ishikawa N, Kimura F (2006). On-pump beating-heart coronary artery bypass grafting for acute myocardialinfarction. Ann Thorac Surg.

[8] Miyahara K, Matsuura A, Takemura H, Saito S, Sawaki S, Yoshioka T (2008). On-pump beating-heart coronary artery bypass grafting after acute myocardial infarction has lower mortality and morbidity. J Thorac Cardiovasc Surg.

[9] Gulcan O, Turkoz R, Turkoz A, Caliskan E, Sezgin AT (2005). Onpump/beating–heart myocardial protection for isolated or combined coronary artery bypass grafting in patients with severe left ventricle dysfunction: assessment of myocardial function and clinical outcome. Heart Surg Forum.

[10] Mizutani S, Matsuura A, Miyahara K, Eda T, Kawamura A, Yoshioka T (2007). On-pump beating-heart coronary artery bypass: a propensity matched analysis. Ann Thorac Surg.

[11] Edgerton JR, Herbert MA, Jones KK, Prince SL, Acuff T, Carter D (2004). On-pump beating heart surgery offers an alternative for unstable patients undergoing coronary artery bypass grafting. Heart Surg Forum.

[12] Takemura H, Watanabe G, Takahashi M, Tomita S, Higashidani K (2003). Beating heart coronary artery bypass grafting: results from 402 patients and the usefulness of gastroepiploic artery composite grafting. Jpn J Thorac Cardiovasc Surg.

[13] Wan IY, Arifi AA, Wan S, Yip JH, Sihoe AD, Thung KH (2004). Beating heart revascularization with or without cardiopulmonary bypass: evaluation of inflammatory response in a prospective randomized study. J Thorac Cardiovasc Surg.

[14] Carrier M, Perrault LP, Jeanmart H, Martineau R, Cartier R, Pagé P (2003). Randomized trial comparing off-pump to on-pump coronary artery bypass grafting in high-risk patients. Heart Surg Forum.

[15] Celik JB, Gormus N, Topal A, Okesli S, Solak H (2005). Effect of off-pump and on-pump coronary artery bypass grafting on renal function. Ren Fail.

[16] Tsai YT, Lin FY, Lai CH, Lin YC, Lin CY, Tsai CS (2012). On-pump beating-heart coronary artery bypass provides efficacious short-and long-term outcomes in hemodialysis patients. Nephrol Dial Transplant.

